# Collecting and analysing cost data for complex public health trials: reflections on practice

**DOI:** 10.3402/gha.v7.23257

**Published:** 2014-02-18

**Authors:** Neha Batura, Anni-Maria Pulkki-Brännström, Priya Agrawal, Archana Bagra, Hassan Haghparast-Bidgoli, Fiammetta Bozzani, Tim Colbourn, Giulia Greco, Tanvir Hossain, Rajesh Sinha, Bidur Thapa, Jolene Skordis-Worrall

**Affiliations:** 1 Institute for Global Health, University College London, London, UK; 2 SNEHA, Mumbai, India; 3 Global Health and Development, London School of Hygiene and Tropical Medicine, London, UK; 4 Health Economics and Systems Group, London School of Hygiene and Tropical Medicine, London, UK; 5 BADAS, Dhaka, Bangladesh; 6 Ekjut, Ranchi, India; 7 MIRA, Kathmandu, Nepal

**Keywords:** cost-effectiveness analysis, cost data, LMIC, multisite, randomised control trials

## Abstract

**Background:**

Current guidelines for the conduct of cost-effectiveness analysis (CEA) are mainly applicable to facility-based interventions in high-income settings. Differences in the unit of analysis and the high cost of data collection can make these guidelines challenging to follow within public health trials in low- and middle- income settings.

**Objective:**

This paper reflects on the challenges experienced within our own work and proposes solutions that may be useful to others attempting to collect, analyse, and compare cost data between public health research sites in low- and middle-income countries.

**Design:**

We describe the generally accepted methods (norms) for collecting and analysing cost data in a single-site trial from the provider perspective. We then describe our own experience applying these methods within eight comparable cluster randomised, controlled, trials. We describe the strategies used to maximise adherence to the norm, highlight ways in which we deviated from the norm, and reflect on the learning and limitations that resulted.

**Results:**

When the expenses incurred by a number of small research sites are used to estimate the cost-effectiveness of delivering an intervention on a national scale, then deciding which expenses constitute ‘start-up’ costs will be a nontrivial decision that may differ among sites. Similarly, the decision to include or exclude research or monitoring and evaluation costs can have a significant impact on the findings. We separated out research costs and argued that monitoring and evaluation costs should be reported as part of the total trial cost. The human resource constraints that we experienced are also likely to be common to other trials. As we did not have an economist in each site, we collaborated with key personnel at each site who were trained to use a standardised cost collection tool. This approach both accommodated our resource constraints and served as a knowledge sharing and capacity building process within the research teams.

**Conclusions:**

Given the practical reality of conducting randomised, controlled trials of public health interventions in low- and middle- income countries, it is not always possible to adhere to prescribed guidelines for the analysis of cost effectiveness. Compromises are frequently required as researchers seek a pragmatic balance between rigor and feasibility. There is no single solution to this tension but researchers are encouraged to be mindful of the limitations that accompany compromise, whilst being reassured that meaningful analyses can still be conducted with the resulting data.

Randomised, controlled trials of public health interventions are often conducted in more than one context to gauge their effectiveness in different populations (1, 2). The trials frequently need to establish whether the interventions are cost-effective and economically feasible at scale (3). Current guidelines for the conduct of cost-effectiveness analysis (CEA) are mainly applicable to facility-based interventions in high-income settings, where the unit of randomisation is the individual and patient level data is readily available (4, 5). These guidelines can be difficult to implement for complex interventions in resource poor countries, when provider cost data are unavailable or of poor quality and the unit of randomisation is the cluster or village. Others have begun to address the difficulties in adapting these guidelines to the low- and middle-income countries’ contexts (6). To our knowledge, however, no study has yet considered the additional complexity of adapting those guidelines to compare findings among resource poor settings. This paper thus aims to enrich the existing literature on adapting guidelines for implementation in resource poor settings. In addition, this paper aims to extend that evidence base to highlight the challenges one might experience when it is necessary to compare findings from trials conducted in different settings.

This paper reflects our own experience working within community-based, cluster-randomised, controlled trials conducted in seven sites, across four low- and middle-income countries, between 1999 and 2011. We describe the challenges of the costing process, the solutions and strategies adopted, and the lessons learned. The intervention we use as our working example involved women’s groups practising participatory learning and action to improve care-seeking and care practices. A meta-analysis demonstrated that women’s groups are associated with a 34% reduction in maternal mortality and a 23% reduction in neonatal mortality (7). This paper focuses only on the process of cost data collection and analysis for these trials and does not discuss the collection and analysis of epidemiological data or cost-effectiveness.

This paper is organised as follows: We first provide a detailed summary of the working example. We then summarise available guidelines for identifying, collecting, and analysing costs, before explaining our difficulties in applying the guidelines. In that explanation, we highlight the particular challenges that may arise when costs must be compared among sites. Finally, we reflect on the key lessons learned, emphasising those that may be general to other studies of this type. Each section begins with a brief review of the literature, followed by reflections on our working example, highlighting the challenges encountered and describing the solutions adopted.

## A working example: multisite community participatory intervention

This paper draws on experience from eight cluster-randomised controlled trials that tested the impact of women’s groups on mortality. The groups had a four-phase participatory learning and action cycle to identify and prioritise problems during pregnancy, delivery, and postpartum; to plan and implement locally feasible strategies to address problems; and to evaluate their activities (7). The unit of randomisation was the village (or slum-cluster in Mumbai). The intervention employed local facilitators (were not health workers) to conduct monthly or fortnightly meetings. The first trial began in 2001 in Makwanpur, Nepal, and seven similar trials were initiated in other sites between 2005 and 2008. Four of the sites adopted a factorial design and tested a second intervention alongside the women’s groups (Table 1). Context-specific health service strengthening was undertaken to a varying degree in the intervention and control areas in all trials. Further details of the trials and their effectiveness have been published elsewhere (8–15).

**Table 1:** Women’s group trials

Location	Organisation	Trial dates (Baseline dates)	Second intervention	Cost analysis
Makwanpur, Nepal	MIRA	2001–2003 (1999–2000)	N/A	Borghi et al. 2005
Dhanusha, Nepal	MIRA	2007–2011 (2006–2007)	Sepsis management	Under progress
Orissa and Jharkhand, India	Ekjut	2005–2008 (2004–2005)	N/A	Tripathy et al. 2010
Mumbai, India	SNEHA	2006–2009 (2005–2006)	N/A	N/A (see More 2012)
Bangladesh	BADAS-PCP	2005–2007 (2003)	Resuscitation training	N/A (see Azad et al. 2010)
Bangladesh (scale-up)	BADAS-PCP	2009–2011 (2008)	N/A	Under progress
Malawi	MaiKhanda	2008–2010 (2007–2008)	Quality improvement	Under progress
Mchinji, Malawi	MaiMwana	2005–2009 (2005)	Volunteer peer counselling	Lewycka et al. 2012

Sources: Manandhar et al. 2004; Borghi et al. 2005; Tripathy et al. 2010; Azad et al. 2010; Houweling et al. 2011; More et al. 2012; Colbourn et al. 2012; Prost et al. 2013.

Despite similarities in design and implementation, there were differences among sites. These differences included the size of the study population, the geography of the study area, baseline neonatal and maternal mortality rates, and participation rates among pregnant women. There were also differences in the characteristics of the implementing organisations, some of which were previously well-established, while others were set up for the purpose of the trial. Such differences can challenge standard guidelines for the collection and analysis of data for a comparative costing. How then can research teams allow for some acceptable level of heterogeneity while maintaining robust comparability across trials? This was the task facing our research team in the context of these trials.

## General considerations for cost data collection

Deciding how costs will be collected necessitates a number of early decisions. Crucial decisions include study perspective, time horizon, how and where data will be sourced, how resource use will be measured and valued, and how accounting costs will be adjusted to arrive at economic costs. Finally, investigators need to consider how to allocate joint costs, how to separate start-up costs from implementation costs, and whether to cost out other trial activities such as monitoring and evaluating research, or cost only the intervention itself. Each of these decisions should be framed within the overall objectives of the study. Our experience with this process is described here.

### Perspective: patient, provider, or societal

A costing can be conducted from the perspective of the patient, provider, purchaser, sponsor, government, or society. Each of these perspectives will bear different costs for the same intervention (16, 17). For example, if a costing is conducted from the provider perspective, costs incurred by patients would not be relevant.

In the context of our trials, two perspectives were appropriate: provider and societal. The provider perspective enables any future provider to weigh the costs and benefits (to their institution) of taking on this intervention against those of other interventions. The societal perspective adds the costs of participation incurred by households to the costs incurred by the provider (3). Because community participatory interventions may produce non-health benefits such as community empowerment (15, 18), a societal perspective would potentially capture health and non-health costs and benefits to the women and their communities, as well as to providers (3, 19).

For several reasons, we elected to adopt a provider perspective. In practice, our trial sites did not have sufficient funds or field experience to collect the household level economic data required for a societal perspective. In addition, the monitoring and evaluation questionnaires were already lengthy, and there was an ethical concern about burdening respondents further. In Dhanusha, Nepal, field teams complained that the monitoring and evaluation questionnaire used in 2010 took a minimum of two hours and two to three visits to complete. In this situation, we might have conducted a separate ‘economics’ survey among a subset of households. However, our surveillance teams were already fully committed, and we could not afford to hire and train new teams. Further, we were reluctant to subject participating households to yet another data collection effort, fearing that this would increase the likelihood of women dropping out of the trial or refusing to participate in future work.

### Time horizon

Interventions are typically evaluated for a period of ‘full scale activity’ to assess the desired effects (20). The World Health Organization recommends a 10-year time horizon to explore the effectiveness of a trial in a single site, and Ramsey et al. (21) recommend that a common time horizon be used for costs and effects. Dhaliwal et al. (6) however, argue that educational programmes can be examined over a one- or two-year time frame because, in this sector, start-up costs are relatively small. These recommendations do not provide specific guidance for costing community-based health interventions in multiple sites.

In our case, and consistent with the approach suggested by Ramsey et al. (21) the costing time horizon was determined by the trial duration, that is, two-and-a-half to three years. To this, we added a start-up period that included activities conducted before the trial began. The end point was the same as that used for calculating programme effect, which was chosen by the team conducting the epidemiological analysis.

### Identifying, measuring, and valuing relevant costs

The literature suggests that cost collection methods depend on the perspective of the CEA. In general, micro-costing tends to be used for studies with a consumer or societal perspective (22, 23), while gross-costing can be used for a provider perspective (24).

Micro-costing records resource use at the patient (cost-object) level and enumerates overheads and capital costs such as office rent and electricity costs separately. These costs are added to measure overall resource use and the total cost of service provision (25, 26). Studies using the micro-costing approach generally source data from administrative databases, self-reported activity logs, time and motion studies, and manager surveys (25–27).

When gross-costing, the total cost of service provision is first calculated at the institutional level and then disaggregated to departments, service units, or patients. Resources are generally assumed to be evenly distributed across end users. For this reason gross-costing is not suitable for services that are not the same for all end users (25, 26, 28). Studies using a gross-costing approach frequently collect retrospective data from accounting databases, tariff books, market prices, or published studies. Because these data are financial or accounting costs, they need to be converted into economic costs. Economic costs include the value of the next best alternative forgone by making a particular choice. In practice, in our sites, this meant that although donated goods and volunteer time appeared as a zero cost in the accounting data, they could not be treated as zero cost items for the purpose of the economic costing. Treating such items as zero cost would result in a downward bias in the cost of the intervention and an upward bias in the cost-effectiveness ratio. This bias is particularly concerning if the goods are not available for free in all settings or if the intervention cannot be delivered using volunteers at scale or in all settings (29). There is no consensus in the literature on the ‘best’ technique for converting accounting costs to economic costs, especially in resource poor settings. One method is to use market prices (28). However, in such settings, market prices may not reflect the true availability of labour and capital owing to the presence of formal and informal labour and capital markets. Market prices may therefore need to be adjusted. A commonly used adjustment technique is shadow pricing, wherein a good or service is assigned a price defined by what an individual must give up to gain an extra unit of the good or service (29, 30). However, such adjustment techniques require context and resource specific calculations, which are time and labour intensive.

We took a provider perspective and collected costs retrospectively using a gross-costing approach (9, 11, 31, 32). Data was sourced from project accounts of the partner institutions in the study countries. Almost every site had either made use of donated goods or volunteers to implement the intervention to some degree. For example, office space in Dhaka, Bangladesh, was provided for free by the government. This space was valued at private sector rental rates. External consultants, who were not paid by the project, provided key input into the start-up phase. For these consultants, we estimated the number of consultancy hours provided and valued this at a rate commensurate with their qualifications and work experience.

In addition to the expected challenges of converting accounting to economic costs, we encountered another practical limitation of project accounts data not commonly documented in the literature. In Bangladesh, we had more than one funder over the full duration of the trial, and each funder kept project accounts using their own method within their own files. Calculating costs for this trial required sourcing cost data from multiple sources including funders no longer involved with the trial. These data then had to be reconciled (i.e. significantly reformatted) before they could be combined. Some of the data was available in hard copy form only and needed to be entered into a spread sheet before we could begin the process of reconciliation. This process was significantly more complex and time consuming than we had anticipated and resulted in delays to the final analysis of cost-effectiveness and to the publication of the main trial paper (10).

### Dealing with joint costs

Joint costs occur when two or more outputs, services, or activities are produced from the same input. Common examples include staff who work in both research and implementation or who oversee several arms of a multi-arm trial. The same could be said for office space housing staff from different projects or having different functions within a single project. If a resource is used by several components of an intervention, or by other programmes that are not part of the intervention, the cost associated with that resource must be allocated proportionally in some way. This is commonly done by assessing time or resource use, measured either by observation or by self-reported methods (17).

For each of our research sites, we needed to distinguish start-up from running costs, women’s groups from health service strengthening, our package of interventions from other intervention(s) implemented by partner institutions, monitoring and evaluation from implementation, research from implementation, and process evaluation from implementation. At all sites, we could directly assign most incurred costs to these categories using appropriate labels in the project accounts. However, for resources that were used across categories, we had little prospective information to use as a basis for the proportional allocation of costs. In some cases, we used information from vehicle logbooks to allocate vehicle purchase, maintenance, and fuel costs as well as drivers’ salary costs (MaiMwana trial) or used fax machine minutes for fax machine costs (Bangladesh trial). Staff time sheets for managerial or administrative staff were available for only two of the trials (Makwanpur and Ekjut trials) (8, 9).

We allocated programme costs to components or categories using a two-step process. First, we directly allocated as many of the staff, material, and capital cost items as possible using the methods described above. Second, the economics team, in consultation with local managerial staff, decided upon a joint cost allocation rule to divide the remainder among the programme components. For example, in MaiMwana (Malawi), 25% of the joint costs were allocated to women’s groups, 25% to monitoring and evaluation, 19% to the peer counselling intervention, 16% to health service strengthening, 10% to process evaluation, and 5% to research. In the Bangladesh scale-up trial, 40% was allocated to women’s groups, 40% to monitoring and evaluation, and 10% to health service strengthening and process evaluation, respectively. Although this resulted in differing allocation rules across sites, the decisions reflected the understanding of actual resource use by local staff who had worked in the implementing organisation during the trial.

### Start-up and implementation costs

Separating start-up and implementation costs is essential if the analysis must tabulate the cost of scaling up an intervention. If, however, an intervention is tested at scale, this may not be necessary. The start-up period is the time between the decision to implement an intervention and delivering it to the first beneficiary (33). Typically, start-up costs include (but are not limited to) costs incurred when recruiting personnel, procuring office space and equipment, and training field workers. Implementation costs are those incurred when the intervention is being implemented and commonly include salaries, transportation costs, overheads, additional materials, and capital goods.

In Nepal (8), we defined start-up costs by type of activity. For example, the design of the pictorial card game was classified as a women’s group start-up cost, and the design of health worker training manuals was classified as a health service strengthening start-up cost (31). However, the field team found this process time intensive and onerous and were reluctant to further participate in the costing. As such, we took a more pragmatic approach to ensure the on-going cooperation of the field teams. We defined a start-up period during which all costs incurred were classified as start-up. The remainder of the costs were classified as implementation costs. In a few instances, costs incurred in the implementation period were classified as start-up if we considered them to be essential for implementation to continue. These included the recruitment and training of new facilitators to replace facilitators who dropped out or moved away. Training of health staff was part of the health service strengthening provided to intervention and control areas and therefore was an implementation cost.

### Research and monitoring and evaluation costs

Once an intervention proves to be effective in a given context, scaling up or replicating that intervention will seldom require the same research activities required for the initial trial. However, some on-going monitoring and evaluation of the intervention is desirable. The economic evaluation of a trial may want to split research from implementation costs and monitoring and evaluation from research costs. The study by Gilson et al. (34) is one of the few to report that research costs were excluded from the cost analysis. We were unable to find any study that distinguished monitoring and evaluation costs from research costs.

In our trials, we defined monitoring and evaluation as all activities conducted by the surveillance team including data officers who conducted data entry and cleaning. It was relatively straightforward to identify the monitoring and evaluation costs because all our sites had a separate monitoring and evaluation team. Thus, all monitoring and evaluation expenses were a separate line item in the project accounts. We defined research costs as activities related to the analysis of data, dissemination of results, and planning of further interventions. Most research activities were conducted by staff from the UK partner institution. However at all sites, local staff also engaged in research activities. Our task was to identify and deduct the latter type of research cost from local project accounts. We identified the cost of meetings and travel for which the primary purpose was research, retrospectively estimated the proportion of time that individual local staff members spent on research each year, and used this proportion to allocate relevant capital costs (e.g. laptops) to research. We also assigned a proportion, based upon staff time use, of all joint costs to research. These research costs were then deducted from the total cost of the project, while the monitoring and evaluation costs were included in the total cost.

Owing to the design of randomised controlled trials, roughly half of monitoring activities are conducted in control areas. It could be argued that this is a research cost that should be subtracted from the total programme cost. We encountered two main difficulties in doing so. The first is that we only had one monitoring and evaluation team per site and therefore, could not clearly differentiate between the costs incurred in intervention versus control areas. Second, the very act of monitoring may have had an impact on the effectiveness of the intervention. Thus, we did not attempt to identify the proportion of monitoring and evaluation activity that was a part of research activity. Instead, we calculated total monitoring and evaluation cost and reported this alongside the intervention implementation cost.

## Complexities of a multisite CEA

Making cost collection decisions, as described earlier, can be challenging in a single setting. Making those decisions when the results must be comparable across multiple settings can be complex in the extreme. In particular, we reflect upon our efforts at collaboration and standardisation.

### Collaborative efforts in cost identification and collection

Many economic evaluations may be conducted by economists based in the evaluating institution and not by staff at trial sites, making a very strong case for collaboration on the CEA of a multisite, randomised, controlled trial. First, collaboration provides opportunities for skill sharing and capacity building, enabling the integration of economic activities into country teams (1). Second, no one economist can be in all sites at all times, and few funders will finance one qualified economist per site! Third, collaboration facilitates data consistency.

We found it useful to establish a key ‘costing contact’ at each site to oversee data collection. This ensured that costs were captured without creating a significant additional burden or without introducing parallel systems that replicated data already being collected for other purposes. These contacts were not all economists or finance personnel but were those interested in the costing process and volunteered to take on the role. Our costing contacts collected data with the help of a custom-made tool described in the next section. Although we found collaboration beneficial overall, we also argue that it was important to have a central lead on the CEA who was able to support the sites, ensure overall consistency, and, ultimately, shape the final analysis.

### Standardisation: designing a cost collection tool

Standardising economic evaluation methods enables users to compare the results of evaluations for different interventions or for a similar intervention in different settings (35). The key ‘standard’ aspects that facilitate comparative analysis are the perspective, measures of outcome or effect, and the definition of inputs and costs (35, 36). If the collection of costs is decentralised to implementing partners who may have varying levels of expertise with economic evaluation, achieving this level of standardisation can be challenging.

During design and implementation of our trials, economists were engaged on an ad hoc basis (9, 21). This resulted in variable progress toward conducting the costing activities across different sites, despite the development and distribution of a manual describing the processes used to collect and analyse the cost data of the first trial (31). To facilitate a comparative costing and to enhance standardisation across our trials, the UK partner institution appointed an economics team in 2009 with overall responsibility for the evaluation of the trials. This led to the design of a common costing tool to be used for each site.

Because the software platform for the costing tool needed to be universally accessible, we avoided the use of specialised statistical and data management software. We used a compatible version of MS Excel, already available and extensively used by all partner institutions. The tool was designed in a way to be easy for a non-economist to understand and use. To ensure that this, the costing contact and other key researchers at the trial site met for a one-day workshop to discuss the purpose of the tool, to learn how to use it, and to agree on definitions of relevant cost categories. Following a first draft of the tool, the UK economics team conducted a series of one-to-one training sessions with key costing contacts at each site. These sessions revealed the degree to which flexibility was needed in the tool to incorporate previously described differences in the structure of the trial, activities, and priorities among the sites. Feedback, opinions, and concerns regarding the use of the tool continue to be shared between the key costing personnel in the UK and the trial sites via e-mail and Skype.

The costing tool will be available in the public domain when the trial data are released. Table 2 presents the structure of the tool. The main worksheets for entering data are staff, material, capital, and joint costs. We also created worksheets that summarise the costs (including a share of joint costs) by programme components (e.g. women’s groups) and that allow additional items to be entered. The final worksheets present results and allow effect data to be entered and cost-effectiveness results to be calculated.

**Table 2:** Costing tool structure

Categories of costs	Data input	Examples of items
Main input categories:		
Staff time and salaries	Salaries and other staff costs. Annual allocation (hours or percentage).	Salaries of programme staff; facilitators; identifiers; volunteers.
Materials and equipment	Purchase year and price. Allocation.	Items that have no resale value after 1 year (e.g. picture cards, questionnaires, HIV testing kits, raincoats).
Capital costs	Purchase date, price, and expected useful life (for items owned). Annual recurrent costs. Annual allocation.	Buildings, vehicles and equipment with an expected useful life >1 year (e.g. laptops, bicycles, cars, photocopiers, office premises).
Joint costs	Summarises share of staff, material, and capital costs already allocated to joint costs. Allows additional items to be input.	Audit fees, licence fees, fuel and travel refunds, training and recruitment costs, meeting costs.
Summary sheets and additional data entry by programme component:		
Women’s group facilitation Health service strengthening or other intervention(s) Monitoring and evaluation Process evaluation Research	Each sheet summarises the share of allocated joint costs and directly allocated staff, material, and capital costs. Additional costs may also be entered.	Fuel and travel refunds, training and recruitment costs, project material development costs, meeting costs.
Results sheets:		
National currency US dollars	Detail of costs with various breakdowns.	
Cost-effectiveness	The user is able to input programme effect data and calculate cost-effectiveness.	

## Discussion

Some of the challenges that we faced while collecting and analysing the cost data across multiple sites may be common to data collection processes for single and multiple site costings. For both, we offer practical solutions for resource poor settings. We do not discuss analytical methods in detail because the use of decision trees or modelling techniques is more closely related to the purpose of economic evaluation.

An important consideration in a costing is the accurate identification and categorisation of different costs. In our trials, this was the case with identifying start-up costs, in particular. Early start-up activities were not always separately identified or even included in project accounts. This is likely to be an issue with all trials where there are multiple funding sources and where interventions are planned before funding begins. We recommend that in such a scenario, the staff involved build a common understanding of cost components and activities and work to reconcile cost data from multiple sources that may be duplicated in different databases or that are available in different formats.

Another very important consideration is whether to exclude research costs from analysis. Separating out the research costs specific to a trial setting from intervention implementation costs is informative if the results are used to estimate scale-up or implementation costs in another setting. We excluded research costs from our analysis but included monitoring and evaluation costs. Although research activities are distinctly necessary in a trial setting, some degree of monitoring and evaluation is desirable when interventions are rolled out. One solution for distinguishing between the two is to identify a minimum amount of cost for monitoring and evaluation and to assign the remainder to research. Another is to identify activities that are clearly research driven, such as control area surveillance, and allocate costs for those activities to research. In the absence of a good allocation method, a conservative approach such as ours is advisable. We simply reported all monitoring and evaluation costs as part of the total project cost. In our trials, we could not rule out the possibility that intense and visible surveillance activities in the community have positive externalities or other unintended positive health outcomes. Excluding monitoring and evaluation in this situation would risk underestimating the total cost and overestimating the cost-effectiveness of the intervention.

To carry out a comparative CEA across multiple sites, it is imperative to collect data that is consistent across all sites. In our experience, collaboration with key costing personnel at each site, led by the UK economics team, proved to be very successful in identifying and collecting provider cost data. The design and use of a custom cost collection tool built on an accessible platform allowed data to be collected in a consistent manner. The tool also allowed a degree of flexibility in the data collection that reflected important site-specific characteristics. The tool and the manner in which it was used by the in-country and UK partners allowed knowledge sharing and capacity building. Further, the collaborative process allowed us to collect the data in a timely manner and to ensure quality.

We found no consensus in the guidelines on how to choose appropriate time horizons or discount rates for costs, especially for community participatory trials in low- and middle-income countries. We believe that the choice of these would depend on the setting of the trial as well as the expectations of scale-ups or replication.

Learning from our experience over a decade, we are able to contribute toward the improvement of cost data collection methodology and systems. This experience has highlighted the importance of planning ahead and of being prepared for the possibility of unexpected events. For example, the start-up of the Dhanusha and Mchinji trials was delayed because of a Maoist uprising and because of unexpected personal events, respectively. Guidelines or recommendations on whether and how to factor in such events into analysis are non-existent. In our case, the delays occurred before implementation. The associated costs were therefore included as part of the start-up costs, effectively becoming one of many factors that contributed to differences in start-up costs across the sites.

Finally, it is worth noting that, given the lifespan of these trials, the staff involved in evaluating the costs and benefits of the interventions has changed. We tried to ensure consistency over time by exchanging information with outgoing researchers, constructing a costing tool that is standardised yet has sufficient flexibility to be adapted by partners for subsequent use, and by training groups of staff on the methods used. The truth is that, with the movement of researchers into and from a project, there will also be a movement of ideas. This presents an opportunity to improve what has been done in the past and poses a challenge to maintain consistency and comparability over time. Trials conducted over multiple sites tend to be large collaborations of implementing and research partners. Each partner will bring their own ideas about what is most important within the research process and how the objectives of the trial may best be achieved. Many of the decisions made regarding a comparative CEA will be made by these different teams. These decisions are likely to reflect necessary compromise in the design, implementation, and analysis.

Our reflections on the practical considerations of collecting cost data for a comparative costing of multiple sites is not an exhaustive list of guidelines or of the challenges faced. It outlines some of the main steps involved in data collection for the economic evaluation of a single site trial and describes our experience putting these steps into practice across multiple sites. In doing so, we provide pragmatic solutions to common problems that may be faced when costing a cluster randomised, controlled, trial in resource poor settings. Further, we hope that the discussion in this paper encourages researchers other than economists to conduct economic evaluations of their interventions.

## References

[1] Meenan RT, Goodman MJ, Fishman PA, Hornbrook MC, O’Keeffe-Rosetti MC, Bachman DJ (2002). Issues in pooling administrative data for economic evaluation. Am J Manag Care.

[2] Schulman K, Burke J, Drummond M, Davies L, Carlsson P, Gruger J (1998). Resource costing for multinational neurologic clinical trials: methods and results. Health Econ.

[3] Drummond MF, Sculpher MJ, Torrance GW, O’Brien BJ, Stoddart GL (2005). Methods for the economic evaluation of health care programmes.

[4] Gyrd-Hansen D, Søgaard J, Kronborg O (1998). Colorectal cancer screening: efficiency and effectiveness. Health Econ.

[5] Whynes D, Neilson AR, Walker AR, Hardcastle JD (1998). Faecal occult blood screening for colorectal cancer: is it cost-effective?. Health Econ.

[6] Dhaliwal I, Duflo E, Glennerster R, Tulloch C (2012). Comparative cost-effectiveness analysis to inform policy in developing countries: a general framework with applications for education. http://www.povertyactionlab.org/publication/cost-effectiveness.

[7] Prost A, Colbourn T, Seward N, Azad K, Coomarasamy A, Copas A (2013). Women’s groups practising participatory learning and action to improve maternal and newborn health in low-resource settings: a systematic review and meta-analysis. Lancet.

[8] Manandhar DS, Osrin D, Shrestha BP, Mesko N, Morrison J, Tumbahangphe KM (2004). Effect of a participatory intervention with women’s groups on birth outcomes in Nepal: cluster-randomised controlled trial. Lancet.

[9] Tripathy P, Nair N, Barnett S, Mahapatra R, Borghi J, Rath S (2010). Effect of a participatory intervention with women’s groups on birth outcomes and maternal depression in Jharkhand and Orissa, India: a cluster-randomised controlled trial. Lancet.

[10] Azad K, Barnett S, Banerjee B, Shaha S, Khan K, Rego AR (2010). Effect of scaling up women’s groups on birth outcomes in three rural districts in Bangladesh: a cluster-randomised controlled trial. Lancet.

[11] Lewycka S, Mwansambo C, Kazembe P, Phiri T, Mganga A, Rosato M (2010). A cluster randomised controlled trial of the community effectiveness of two interventions in rural Malawi to improve health care and to reduce maternal, newborn and infant mortality. Trials.

[12] Houweling TA, Azad K, Younes L, Kuddus A, Shaha S, Haq B (2011). The effect of participatory women’s groups on birth outcomes in Bangladesh: does coverage matter? Study protocol for a randomized controlled trial. Trials.

[13] Shrestha BP, Bhandari B, Manandhar DS, Osrin D, Costello A, Saville N (2011). Community interventions to reduce child mortality in Dhanusha, Nepal: study protocol for a cluster randomized. Trials.

[14] More NS, Bapat U, Das S, Alcock G, Patil S, Porel M (2012). Community mobilization in Mumbai slums to improve perinatal care and outcomes: a cluster randomized controlled trial. PLoS Med.

[15] Colbourn T, Nambiar B, Bondo A, Makwenda C, Tsetekani E, Makonda-Ridley A (2013). Effects of quality improvement in health facilities and community mobilization through women’s groups on maternal, neonatal and perinatal mortality in three districts of Malawi: MaiKhanda, a cluster randomized controlled effectiveness trial. Int Health.

[16] Gold MR, Siegel JA, Russell LB, Weinstein MC, Gold MR, Siegel JA, Russell LB, Weinstein MC (1996). Estimating costs in cost-effectiveness analysis. Cost-effectiveness in health and medicine.

[17] Mogyorosy Z, Smith M (2005). The main methodological issues in costing health care services. CHE Research Paper 7.

[18] Rosato M, Laverack G, Grabman LH, Tripathy P, Nair N, Mwansambo C (2008). Community participation: lessons for maternal, newborn, and child health. Lancet.

[19] Weinstein MC, Siegel JE, Gold MR, Kamlet MS, Russell LB (1996). Recommendations of the panel on cost-effectiveness in health and medicine. JAMA.

[20] Edejer TTT (2003). Making choices in health: WHO guide to cost effectiveness analysis.

[21] Ramsey S, Willke R, Briggs A, Brown R, Buxton M, Chawla A (2005). Good research practices for cost-effectiveness alongside clinical trials: The ISPOR Force Report. Value Health.

[22] Muralikrishnan R, Venkatesh R, Venkatesh P, Frick K (2004). Economic cost of cataract surgery procedures in an established eye care centre in Southern India. Neuro-Ophthalmology.

[23] Henry SG, Ness RM, Dittus RS (2007). A cost analysis of colonoscopy using microcosting and time-and-motion techniques. J Gen Intern Med.

[24] Yukich JO, Lengeler C, Tediosi F, Brown N, Mulligan JA, Chavasse D (2008). Costs and consequences of large-scale vector control for malaria. Mala J.

[25] Edbrooke DL, Hibbert CL (1999). Cost determinants and economic assessment in the critical care setting. Curr Opin Crit Care.

[26] Waters HR, Hussey P (2004). Pricing health services for purchasers—a review of methods and experiences. Health Policy.

[27] Zelman WN (1987). Cost per unit of service. Eval Program Plann.

[28] Beecham J, Knapp MRJ (1995). Collecting and estimating costs. The economic evaluation of mental health care.

[29] Walker D (2001). How to do (or not to do) … Cost and cost-effectiveness guidelines: which ones to use?. Health Policy Plan.

[30] Hutton G, Baltussen R (2005). Cost valuation in resource-poor settings. Health Policy Plan.

[31] Borghi J, Thapa B, Osrin D, Jan S, Morrison J, Tamang S (2005). Economic assessment of a women’s group intervention to improve birth outcomes in rural Nepal. Lancet.

[32] Fottrell E, Azad K, Kuddus A, Younes L, Shaha S, Nahar T (2013). The effect of increased coverage of participatory women’s groups on neonatal mortality in Bangladesh: a cluster randomized trial participatory women’s groups in Bangladesh. JAMA Pediatr.

[33] Johns B, Baltussen R, Hutubessy R (2003). Programme costs in the economic evaluation of health interventions. Cost Eff Resour Alloc.

[34] Gilson L, Mkanje R, Grosskurth H, Mosha F, Picard J, Gavyole A (1997). Cost-effectiveness of improved treatment services for sexually transmitted diseases in preventing HIV-1 infection in Mwanza Region, Tanzania. Lancet.

[35] Drummond M, Brandt A, Luce B, Rovira J (1993). Standardizing methodologies for economic evaluation in health care: practice, problems, and potential. Int J Technol Assess Health Care.

[36] Russell LB (1986). Is prevention better than cure?.

